# Efficacy and safety of PARP inhibitors monotherapy or combination therapy with anti-angiogenics in ovarian cancer: a network meta-analysis

**DOI:** 10.3389/fonc.2025.1713163

**Published:** 2026-01-26

**Authors:** Yuan Liu, Tongtong Ren, Xinchun Wang, Yuqi Wang, Lisha Tang, Wenjiang Cao

**Affiliations:** 1 The First Affiliated Hospital Shihezi University, Shihezi, Xinjiang, China; 2 Pharmacy of College, Shihezi University, Shihezi, Xinjiang, China; 3 Lianyungang Maternal and Child Health Hospital Affiliated to Kangda College of Nanjing Medical University, Lianyungang, Jiangsu, China; 4 General Hospital of Xinjiang Military Region of the People’s Liberation Army of China, Urumqi, Xinjiang, China

**Keywords:** antiangiogenic agents, efficacy, ovarian cancer, PARP inhibitors, safety

## Abstract

**Background:**

The incidence of ovarian cancer ranks second only to cervical cancer and uterine cancer, but its mortality rate is the highest. Searching for effective and safe PARP inhibitors - antiangiogenic drugs combined treatment for ovarian cancer is a new approach.

**Method:**

Conducted a comprehensive search in the authoritative databases, with the search period ranging from the establishment of these databases until December 2024. And conducted a Bayesian network meta-analysis using R 4.3.1 and Stata 16.0.The primary endpoint was progression-free survival (PFS), and the secondary endpoint was adverse events (AEs) (≥grade 3).

**Results:**

The analysis ultimately incorporated 15 RCTs from 18 publications, involving 6,416 patients and evaluating nine distinct treatment regimens. All PARPi monotherapies and combination therapies demonstrated significant PFS improvement versus placebo (P ≤ 0.05). Compared with niraparib alone(HR = 2.85; 95%CI:1.2-6.79), niraparib+bevacizumab showed significant difference in improving PFS in ovarian cancer patients (P ≤ 0.05). olaparib+cediranib had significant difference in improving PFS compared with olaparib(HR = 1.36; 95%CI:1.06-2.26) (P ≤ 0.05). Besides, niraparib+bevacizumab ranked first in improving PFS, followed by olaparib+ cediranib. In terms of safety, there was no statistically significant difference in AEs (grade≥3) between different PARP inhibitors or in combination with antiangiogenic agents for ovarian cancer.

**Conclusion:**

Current evidence indicates that the combination of PARPi and anti-angiogenic drugs in the treatment of ovarian cancer is superior to PARPi monotherapy. Niraparib combined with bevacizumab may show the most optimal effect in improving PFS, and it might be a better combined treatment option. In monotherapy, senaparib has shown good efficacy. The incidence of AEs of various PARPi is similar, but the incidence of adverse reactions is relatively high when used in combination mode. A reasonable treatment plan should be selected based on the individual conditions of patients.

**Systematic Review Registration:**

https://www.crd.york.ac.uk/prospero/, identifier CRD 420251003413.

## Introduction

Ovarian cancer (OC), a malignancy arising from germ cells, ranks as the sixth most common cancer and fifth leading cause of cancer mortality in developed nations. Annually diagnosed in over 20,000 women, it accounts for 67% of gynecological cancer deaths, with higher incidence in North America/Western Europe than Africa/Asia (1). Predominantly affecting postmenopausal women, OC exhibits nonspecific symptoms and delayed diagnosis, with most cases identified at advanced stages (III/IV) (2). Standard treatment combines surgery and chemotherapy, yet 70% of patients relapse within 3 years, developing multidrug resistance with progressively shorter remissions (3). Five-year survival rates remain critical at 36% (stage III) and 17% (stage IV) (4).

DNA damage occurs persistently throughout life, activating appropriate repair mechanisms. However, ovarian cancer (OC) patients frequently exhibit genetic defects in these repair pathways. Common DNA lesions include single-strand breaks (SSBs) primarily repaired through base excision repair (BER), and double-strand breaks (DSBs) predominantly addressed by homologous recombination (HR) or non-homologous end joining (NHEJ) (5). Poly(ADP-ribose) polymerase (PARP), a crucial enzyme in DNA repair, mediates approximately 90% of DNA damage responses (6). The HR pathway depends on homologous recombination repair (HRR) genes, particularly BRCA1 and BRCA2. Approximately 25% of newly diagnosed OC patients exhibit BRCA mutations. Deficient HR-mediated repair (HRD) occurs in about 50% of high-grade serous ovarian carcinoma (HGSOC) cases, representing the most prevalent OC subtype with compromised DNA damage response capabilities (7).

PARP inhibitors (PARPi) exert antitumor effects by targeting PARP1/2 proteins to disrupt single-strand break repair, inducing synthetic lethality in BRCA1/2-mutated or HRD-positive ovarian cancer (OC) cells (8). Clinical evidence demonstrates PARPi’s potential as first-line maintenance therapy, significantly improving progression-free survival (PFS) and prognosis in OC patients (9). Currently, six PARPi have received FDA approval for cancer treatment: olaparib, rucaparib, niraparib, talazoparib, fuzuloparib, and pamiparib (10). Among these, olaparib, rucaparib, and niraparib are specifically approved by both FDA and EMA for epithelial ovarian cancer (11, 12). However, recent studies indicate limitations in PARPi’s ability to enhance long-term survival rates, with emerging drug resistance in BRCA-mutant tumors prompting the need for novel therapeutic strategies (13).

Anti-angiogenic agents represent a promising strategy to overcome PARPi resistance in ovarian cancer (OC) (14). As a key driver of OC progression and malignant ascites formation, VEGF/VEGFR signaling has become a primary therapeutic target. Current anti-angiogenic therapies mainly include VEGF inhibitors (e.g., bevacizumab), tyrosine kinase inhibitors (pazopanib, nintedanib), and angiopoietin inhibitors. Emerging evidence reveals synergistic mechanisms between PARPi and anti-angiogenic therapy: 1) Anti-angiogenic agents induce tumor hypoxia by reducing neovascularization, thereby downregulating homologous recombination repair factors and creating BRCA-like vulnerability (12, 14); 2) PARPi demonstrates potential to mitigate VEGF inhibitor-associated vascular toxicity, though paradoxical pro-angiogenic effects via VEGF-A upregulation require further investigation (15). Notably, clinical trials have validated the enhanced anti-tumor efficacy of this combination strategy (16).

The combination of PARP inhibitors and anti-angiogenic agents has emerged as a promising first-line therapeutic approach for advanced and recurrent ovarian cancer, demonstrating benefits in enhancing survival outcomes and reducing adverse events. The current meta-analyses reporting on the combined treatment of ovarian cancer with PARPi have not clearly identified the differences in the therapeutic effects of specific combination drugs, nor have they reached a definite conclusion on the differences in safety (5, 17–19). This network meta-analysis systematically evaluates these treatment regimens, aiming to provide evidence-based recommendations for clinical decision-making in ovarian cancer management.

## Methods

### Registration

The study types selected for this meta-analysis were retrospective studies. We followed the “Preferred reporting items for systematic reviews and meta‐analyses” (PRISMA) statement for reporting of this meta-analysis. The study has been registered at PROSPERO (CRD 420251003413).

### Search strategy

Relevant studies on advanced ovarian cancer, recurrent ovarian cancer, PARP inhibitors, and anti-angiogenic drugs were retrieved from PubMed, Embase, Web of ScienceThe Cohrane Library from the database establishment to December 2024. The medical subject headings (MeSH) containing these terms were searched in the PubMed database in accordance with PICOS principles (20). A combination of subject words and free words was used, in which the search subject words included “ovarian cancer”, “PARP inhibitor”, “PARP inhibitor combined with anti-angiogenic drugs”, and the free words included: “Ovarian Neoplasms”, “Poly(ADP-ribose) Polymerase Inhibitors”, “PARP inhibitors”, “olaparib”, “niraparib”, “rucaparib”, “veliparib” and “senaparib” (21).

### Study eligibility criteria

A dual-independent screening process was implemented in accordance with the PICOS framework (Population/Intervention/Comparison/Outcome/Study design). Inclusion criteria (22): (1) Histologically confirmed ovarian cancer (regardless of BRCA/HRD status); (2) PARPi-containing regimens (monotherapy or combination therapy); (3) Reporting efficacy/safety endpoints (PFS, OS, or grade ≥3 AEs); (4) RCTs or high-quality observational studies. Exclusion criteria (23): (1) Studies with incomplete outcome data (>20% missing); (2) Non-ovarian cancer populations; (3) Early-phase trials (phase I) or single-arm studies; (4) Secondary publications/non-clinical research (conference abstracts, meta-analyses, animal studies). Study prioritization for duplicated records favored those with the longest follow-up or most comprehensive datasets.

### Data extraction and bias risk assessment

This network meta-analysis adheres to the PRISMA guidelines throughout its processes. Two researchers independently extracted and tabulated data from eligible studies, compiling detailed information including study titles, interventions, sample sizes, clinical characteristics of the patients, and outcome (19, 20). The primary endpoint in this network meta-analysis is progression-free survival (PFS) with hazard ratios and corresponding 95% confidence intervals, assessed from randomization to the time of tumor progression or death according to RECIST (19). Adverse events (AEs) are considered as secondary endpoints. The risk of bias in the included randomized controlled trials (RCTs) was evaluated by two assessors using the Cochrane Handbook’s risk of bias assessment tool for RCTs, and a quality assessment graph was created using RevMan 5.3. After independent literature searches, screenings, quality assessments, and data extractions by two researchers, cross-verification was conducted, with any disagreements arbitrated by a third senior researcher (11, 19).

### Statistical analysis

For survival data and dichotomous variables, hazard ratios (HR) were employed as effect size measures with corresponding 95% confidence intervals (CI). Bayesian network meta-analysis was implemented through the gemtc, coda, and rjags packages within the R statistical environment. Treatment efficacy and safety profiles were ranked according to surface under the cumulative ranking curve (SUCRA) values, where higher SUCRA scores indicated superior therapeutic performance and the higher incidence of adverse events (11). The analytical workflow was further validated using Stata 16.0 software. Three essential packages were sequentially installed through command-line operations: “findit mvmeta”, “findit metan” and “findit network”. Processed datasets were subsequently imported into the software environment to generate comprehensive analytical outputs (20). Meanwhile, considering the heterogeneity of the included studies, we used I^2^ and χ^2^ tests to evaluate whether there was heterogeneity in this study. When I^2^ was less than 50% or the p-value was greater than 0.1, we adopted the fixed-effect model; otherwise, we used the random-effect model. We will also consider the influence of factors on the treatment outcome, and conduct subgroup analyses.

## Results

### Literature search and screening outcomes

**Figure 1:**
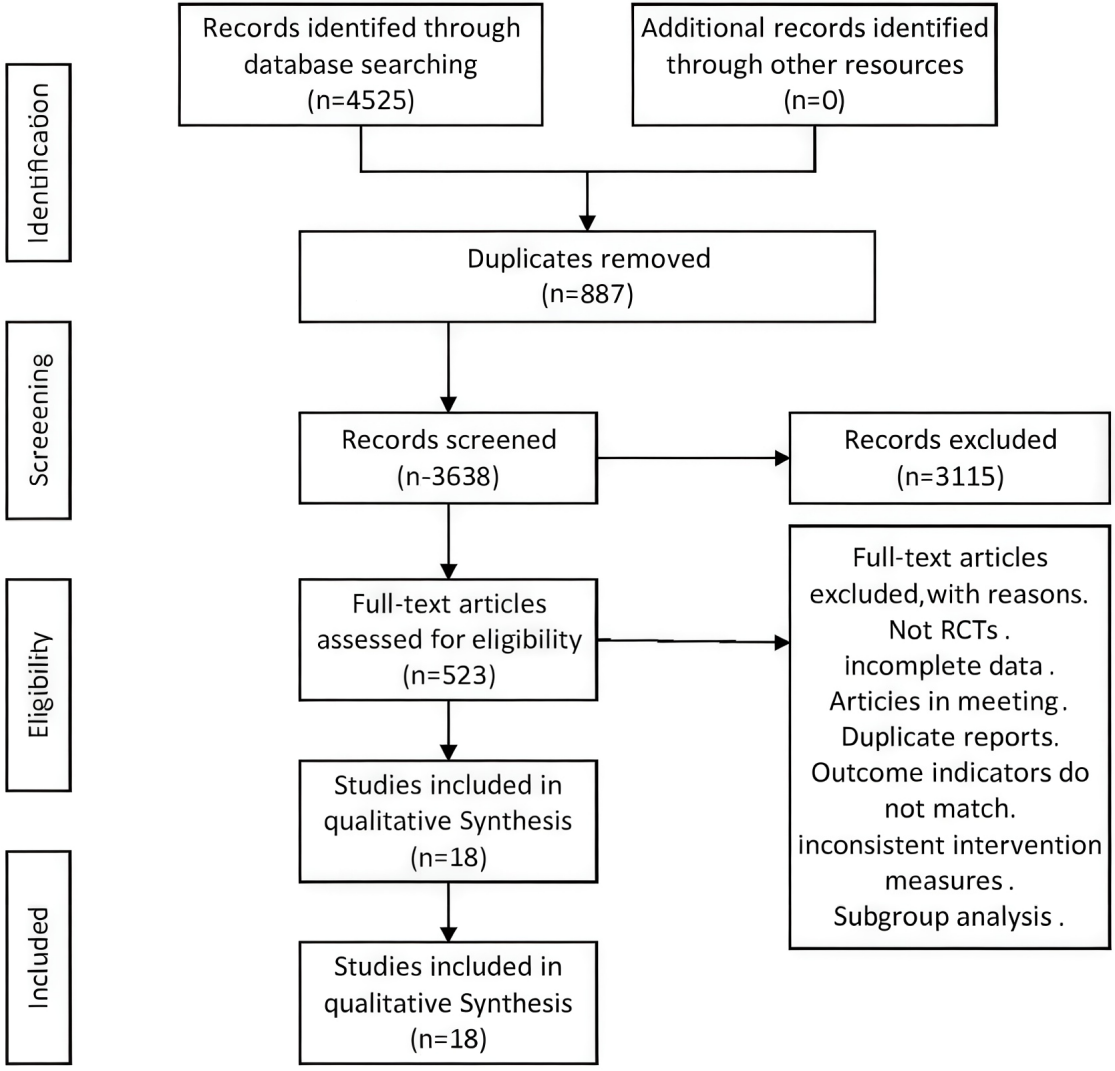
Flow diagram of the systematic search and article selection process.

This meta-analysis initially identified 4,525 potentially relevant articles, with 3,638 remaining after deduplication. Following title and abstract screening, 3,115 records were further excluded. Full-text reviews of the remaining 523 articles led to the exclusion of 505 publications due to the following reasons: non-randomized controlled trials, incomplete datasets, conference abstracts, or interventions inconsistent with inclusion criteria. Ultimately, 18 studies involving 6,416 ovarian cancer patients across disease stages were included for quantitative synthesis. The literature selection flowchart is presented in **Figure 1**. With baseline characteristics of the included studies summarized in **Table 1**, and the data of the subjects included in the table are genuine and there is no overlap among them.

**Table 1:** Characteristics of the studies included in the meta-analysis.

NO	Study	Type	Year	Treatment	No. of patients	PFS	AEs
1	NCT01844986 (SOLO1/GOG 3004) (29)	Recurrent Ovarian Carcinoma	2023	Olaparib Placebo	260 131	HR=0.37; 95%CI:0.28-0.48	T:13.74% C:21.15%
2	NCT01847274, (ENGOT-OV16/NOVA) (30)	Advanced Ovarian Cancer	2019	Niraparib Placebo	367 179	HR=0.35; 95%CI: 0.18-0.71	T:5.59% C:33.79%
3	NCT02655016 (PRIMA/ENGOT-OV26/GOG-3012) (31)	Recurrent Ovarian Cancer	2023	Niraparib Placebo	487 246	HR=0.66; 95%CI:0.56-0.79;	T:22.95% C:72.93%
4	NCT02855944 (ARIEL4) (32)	Newly Diagnosed Ovarian Cancer	2022	Rucaparib Chemotherapy	232 113	HR=0.67; 95%CI:0.52-0.86	T:11.50% C:26.72%
5	NCT02354131, (NSGO-AVANOVA2/ENGOT-ov24) (33)	Relapsed Ovarian Cancer	2019	Niraparib+Bevacizumab Niraparib	48 49	HR=0.35; 95% CI:0.21-0.57	T:44.90% C:64.58%
6	NCT01847274, (ENGOT-OV16/NOVA) (34)	Platinum-Sensitive, Recurrent Ovarian Carcinoma	2016	Niraparib Placebo	367 179	HR=0.4; 95% CI:0.34-0.61	T:5.68% C:33.79%
7	NCT03522246; (ATHENA-MONO/GOG-3020/ENGOT-ov45) (35)	Platinum-Sensitive, Recurrent Ovarian Cancer	2022	Rucaparib Placebo	427 111	HR=0.47; 95% CI:0.31-0.72	T:0.90% C:14.52%
8	ISRCTN14784018 (OCTOVA) (36)	Newly Diagnosed Ovarian Cancer	2024	Olaparib+Cediranib Olaparib	47 46	HR=0.73; 60%CI:0.59-0.89	T:4.35% C:14.89%
9	NCT00628251 (SOLO3) (37)	Platinum-Resistant Ovarian Cancer	2020	Olaparib Chemotherapy	151 72	HR=0.62; 95%CI:0.43-0.91	T:18.06% C:23.18%
10	NCT01874353 (SOLO2/ENGOT- Ov21) (38)	Platinum-Sensitive, Relapsed Ovarian Cancer	2017	Olaparib Placebo	196 99	/	T:2.02% C:20.92%
11	NCT01874353 SOLO2/ENGOT-Ov21) (39)	Platinum-Sensitive, Relapsed Ovarian Cancer	2022	Olaparib Placebo	205 80	HR=0.43; 95%CI:0.24-0.81	/
12	NCT02655016 (PRIMA/ENGOT-OV26/GOG-3012) (40)	Platinum-Sensitive, Recurrent Ovarian Cancer	2024	Niraparib Placebo	190 99	HR=0.61; 95%CI:0.47-0.81	T:1.01% C:45.79%
13	NCT04169997 (41)	Advanced Ovarian Cancer	2024	Senaparib placebo	271 133	HR=0.43; 95%CI:0.32-0.58	T:3.76% C:27.68%
14	NCT01968213, (ARIEL3) (42)	Advanced Ovarian Cancer	2020	Rucaparib Placebo	375 189	HR=0.66; 95%CI:0.53-0.82	T:10.58% C:22.13%
15	NCT03863860 (FZOCUS-2) (43)	Platinum-Sensitive, Recurrent Ovarian Cancer	2022	Fuzuloparib Placebo	167 85	HR=0.46; 95% CI:0.29-0.74	T:2.35% C:7.19%
16	NCT03709316 (44)	Platinum-Sensitive, Recurrent Ovarian Cancer	2023	Niraparib placebo	255 128	HR=0.45; 95%CI: 0.34-0.60	T:17.97% C:54.51%
17	NCT01116648 (45)	Newly Diagnosed Advanced Ovarian Cancer:	2014	Cediranib+Olaparib Olaparib	44 46	HR=0.42; 95%CI: 0.23-0.76	T:10.87% C:68.18%
18	NCT02446600. (NRG-GY004) (46)	Platinum-Sensitive, Recurrent Ovarian Cancer	2022	Cediranib+Olaparib Chemotherapy	172 173	HR=0.86; 95% CI: 0.66 -1.10	T:9.25% C:23.26%

### Risk of bias assessment in included studies

**Figure 2:**
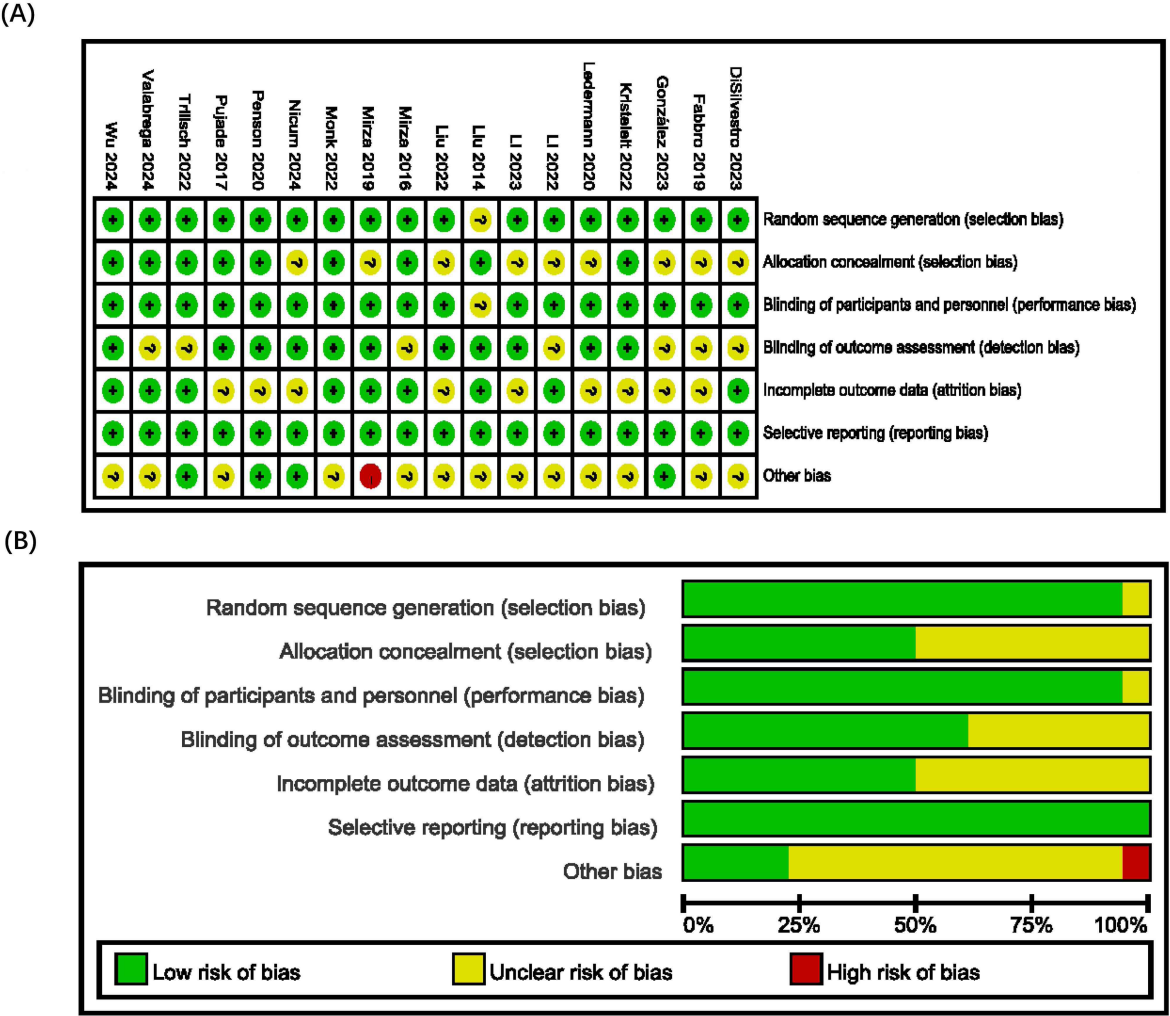
Risk of bias summary graph **(A)** and RCTs literature bias risk assessment **(B)**.

This meta-analysis included a total of 18 papers, with the basic characteristics of the studies presented in **Table 1**. The quality of the included studies was evaluated using the Cochrane Collaboration’s tool, which assessed the risk of bias in the literature. All studies described the generation of random sequences and allocation concealment. Nearly all studies were deemed to have a low risk of selection bias or an unclear risk. Regarding the reporting of outcomes and other biases, the majority of studies were classified as low risk or unclear, with only a few being considered high risk. The risk of bias is illustrated in **Figure 2**. This indicates that the overall quality of the included literature is high.

### Evaluation of therapeutic effect of each treatment measure

**Figure 3:**
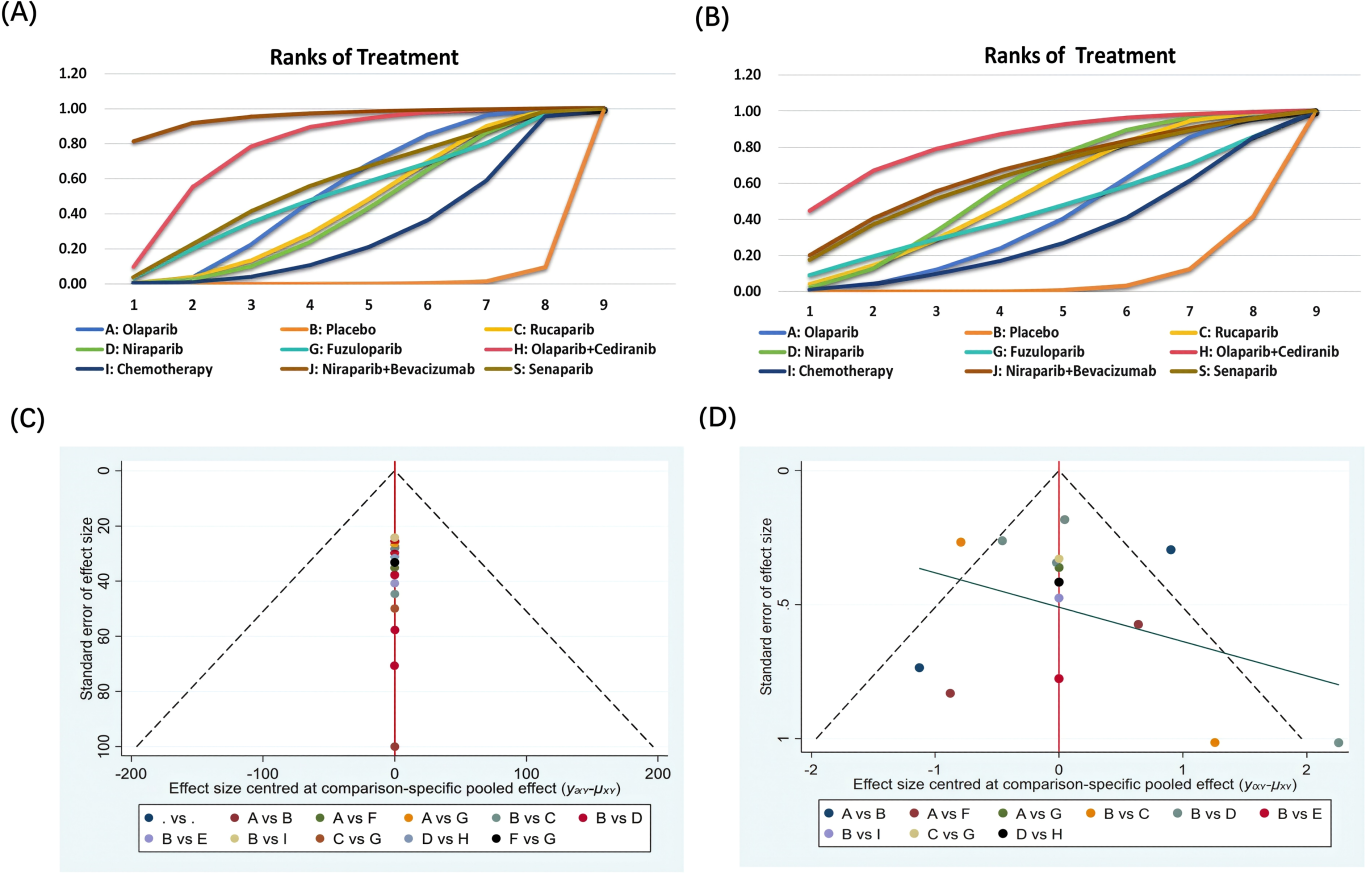
Network diagram and Forest plot of efficacy and safety analysis. **(A, B)** progression-free survival (PFS); **(B, D)** incidence of grade≥3 adverse events (AEs). Annotation: A=Olaparib; B=Placebo; C=Rucaparib; D=Niraparib; G= Fuzuloparib; H=Olaparib+ Cediranib; I=Chemotherapy;J=Niraparib+Bevacizumab; S=Senaparib **(C, D)** Annotation: A=Olaparib; B=Placebo; C=Rucaparib; D=Niraparib; G= Fuzuloparib; H=Olaparib+ Cediranib; I=Chemotherapy;J=Niraparib+Bevacizumab; S=Senaparib.

A total of 17 articles reported PFS outcomes, and a total of 9 treatment options were included in the mesh meta-analysis (**Figure 3A**). The network diagram and pairwise comparison forest diagram of PFS result indicators are shown in **Figure 3A** and **Figure 3C**. By league table analysis, the results showed that compared with the placebo, PARPi monotherapy or combined with anti-angiogenic drugs demonstrated significant differences in improving the PFS of ovarian cancer patients (P ≤ 0.05). For instance, olaparib (HR = 0.46, 95%CI: 0.27-0.76), rucaparib (HR = 1.99, 95%CI: 1.22-3.28), niraparib (HR = 1.94, 95%CI: 1.39-2.8), olaparib+cediranib (HR = 2.98, 95%CI: 1.96-5.98), and niraparib+bevacizumab (HR = 5.55, 95%CI: 2.2-14.21) were all compared with placebo(**Table 2A**) (24). Besides, Compared with olaparib alone(HR = 1.36; 95%CI: 1.06-2.26), olaparib+cediranib had significant difference in improving PFS (P ≤ 0.05). Compared with niraparib alone(HR = 2.85; 95%CI: 1.2-6.79), niraparib+bevacizumab had a significant difference in improving PFS (P ≤ 0.05) (**Table 2**). In addition, compared with chemotherapy, olaparib+cediranib (HR = 0.57; 95%CI:0.32-0.98), there were significant differences in PFS index (P ≤ 0.05).

**Table 2:** League tables for different treatment options (progression-free survival and incidence of grade≥3 adverse events).

Olaparib								
**0.46 (0.27, 0.76)**	Placebo							
0.91 (0.49, 1.68)	**1.99 (1.22, 3.28)**	Rucaparib						
0.89 (0.48, 1.68)	**1.94 (1.39, 2.8)**	0.98 (0.54, 1.8)	Niraparib					
0.99 (0.37, 2.65)	**2.17 (1.04, 5.02)**	1.09 (0.41, 2.87)	1.12 (0.44, 2.74)	Fuzuloparib				
**1.36 (1.06, 2.26)**	**2.98 (1.56, 5.98)**	1.5 (0.75, 3.12)	1.53 (0.73, 3.31)	1.37 (0.48, 4.12)	Olaparib+ Cediranib			
0.77 (0.45, 1.31)	1.68 (0.91, 3.18)	0.85 (0.46, 1.55)	0.87 (0.42, 1.76)	0.78 (0.27, 2.24)	**0.57 (0.32, 0.98)**	Chemotherapy		
2.53 (0.88, 7.43)	**5.55 (2.2, 14.21)**	2.79 (0.97, 8.06)	**2.85 (1.2, 6.79)**	2.55 (0.74, 9.17)	1.86 (0.58, 5.8)	3.29 (1.07, 10.14)	Niraparib+Bevacizumab	
1.06 (0.42, 2.67)	**2.33 (1.08, 5.03)**	1.17 (0.47, 2.89)	1.2 (0.51, 2.73)	1.07 (0.34, 3.33)	0.78 (0.28, 2.1)	1.38 (0.51, 3.67)	0.42 (0.12, 1.38)	Senaparib
Olaparib								
3.47 (0.63, 24.7)	Placebo							
0.67 (0.06, 6.43)	0.19 (0.02, 1.06)	Rucaparib						
0.57 (0.06, 5.71)	**0.16 (0.04, 0.6)**	0.85 (0.09, 9.38)	Niraparib					
0.94 (0.02, 34.83)	0.27 (0.01, 5.41)	1.41 (0.04, 53.9)	1.65 (0.05, 49.03)	Fuzuloparib				
0.19 (0.02, 1.46)	**0.05 (0, 0.76)**	0.28 (0.01, 6.6)	0.33 (0.01, 7.05)	0.2 (0, 12.66)	Olaparib+Cediranib			
1.46 (0.15, 13.79)	0.42 (0.03, 4.08)	2.18 (0.24, 21.41)	2.57 (0.15, 40.11)	1.55 (0.03, 80.79)	7.82 (0.36, 166.93)	Chemotherapy		
0.39 (0.01, 13.82)	0.11 (0, 2.14)	0.58 (0.02, 21.91)	0.69 (0.05, 10.36)	0.42 (0.01, 33.24)	2.11 (0.03, 128.64)	0.27 (0.01, 13.37)	Niraparib+Bevacizumab	
0.43 (0.02, 13.8)	0.12 (0.01, 2.08)	0.64 (0.03, 22.33)	0.75 (0.04, 19.35)	0.46 (0.01, 34.5)	2.3 (0.05, 135.74)	0.29 (0.01, 13.85)	1.09 (0.02, 77.53)	Senaparib

Significant values are in bold. (A) progression-free survival (PFS); (B) incidence of grade≥3 adverse events (AEs).

**Figure 4:**
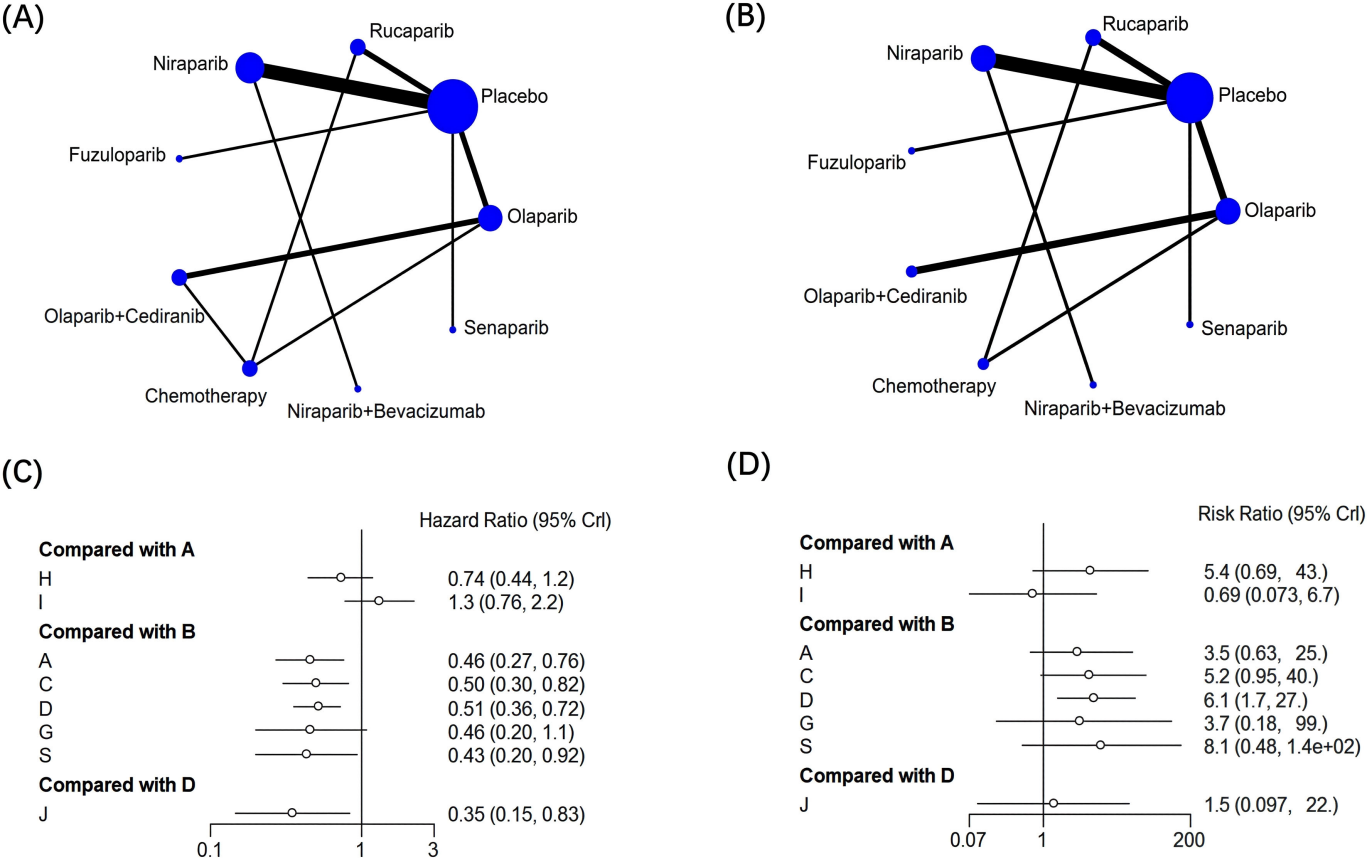
Curve diagram of SUCRA of outcome indicators and Funnel plots for publication bias. **(A, C)** progression-free survival (PFS); **(B, D)** incidence of grade≥3 adverse events (AEs). Annotation: A=Olaparib; B=Placebo; C=Rucaparib; D=Niraparib; G= Fuzuloparib; H=Olaparib+ Cediranib; I=Chemotherapy;J=Niraparib+Bevacizumab; S=Senaparib.

According to the SUCRA ranking probability graph calculated by R software, all outcome indicators were ranked to show the probability of each treatment measure in the corresponding rank. The greater the cumulative SUCRA ranking probability, the greater the possibility of ranking the therapeutic effect of the treatment plan. For the ranking of SUCRA values of PFS outcome indicators in ovarian cancer patients (**Figure 4A**), niraparib+bevacizumab is most likely to rank first among the 9 treatment options for prolonging PFS in OC patients. Through SUCRA sequencing result analysis, In terms of PFS, niraparib+bevacizumab(95.5%)>olaparib+cediranib(78.2%)> senaparib(56.8%)>olaparib(52.8%)>fuzuloparib(51.4%)>rucaparib(44.1%)> niraparib(41.4%) > chemotherapy(28.4%) >placebo(1.3%) (**Figure 4A**).

### Comparison of safety evaluation and incidence of AEs (≥grade 3)

A total of 15 articles reported AEs outcomes, including nine interventions (**Figure 3B**). The network diagram and pairwise comparison forest diagram of outcome indicators of adverse event incidence are shown in **Figures 3B, D**. The results showed that, the incidence of adverse events was significantly higher in the niraparib compared to the placebo (RR = 0.16; 95% CI: 0.04 - 0.6). Similarly, olaparib+cediranib compared to the placebo (RR = 0.05, 95%CI: 0-0.76) also had a significantly higher incidence of adverse events (P ≤ 0.05) (**Table 2**). AEs (≥grade 3) occurs between PARP inhibitors (senaparib, olaparib, fuzuloparib, rucaparib, niraparib) 、olaparib+cediranib、niraparib+bevacizumab and chemotherapy showed no statistically significant difference (**Table 2**), indicating that there was no significant difference in the probability of grade 3 and grade 4 adverse events between these treatments (P > 0.05).

Ranking the SUCRA values derived from the probability of AEs (≥grade 3) in ovarian cancer patients (**Figure 4B**), olaparib+cediranib is most likely to rank first among all treatment options in the probability of adverse events in OC patients, and its SUCRA value is 83.2%. The second is olaparib+cediranib. In terms of the probability of adverse events, niraparib+bevacizumab (83.2%) >olaparib+cediranib(66.1%) >senaparib(63.8%)>niraparib(58.5%)>rucaparib(54.5%)>fuzuloparib(44.8%)>olaparib(40.9%)>chemotherapy(30.8%) >placebo(7.34%) (**Figure 4B**).

### Subgroup analysis

Based on the existing data from 18 literatures, we separately counted the possible subgroups that could be analyzed, including the patient’s age, metastasis status, treatment methods, and tumor mutation status. Due to insufficient data volume, we only conducted a PFS network meta-analysis for the subgroups. The results showed that in different subgroups, compared with the use of PARP inhibitors alone, combined anti-angiogenic drugs treatment improved PFS in different subgroups, and the differences were statistically significant (P < 0.05), regardless of BRCA/HRD status, the SUCRA analysis results are consistent with the whole. Among the population with BRCA mutations, niraparib+bevacizumab had significant difference in improving PFS compared with niraparib (HR = 2.04; 95%CI: 1.91-4.57) and placebo (HR = 5.75; 95%CI: 2.22-14.16) (P ≤ 0.05). Furthermore, whether using PARP inhibitors as a single agent or in combination therapy, there are significant differences in PFS compared to the placebo group(P ≤ 0.05).For the same PARP inhibitors combined with anti-angiogenic drugs treatment strategy, there was no statistically significant difference in the analysis of other subgroups. The heterogeneity of each subgroup was relatively small (I^2^ < 50%).

### Heterogeneity and inconsistency assessment

We employed a random effects model to assess the heterogeneity of progression-free survival (PFS) and adverse events (AEs). In the overall PFS analysis and comparison, we observed moderate heterogeneity, with an I^2^ value of 50.2%. Furthermore, in the AEs analysis and comparison, we discovered a relatively high heterogeneity (I^2^ value 80.3%). Due to the high heterogeneity among the studies, the results of the random effects model only reflected the average trend of the effect size, with greater uncertainty. In the PFS outcome, the heterogeneity test showed that χ²= 34.18, df = 17, P = 0.0035. After stratification by “ tumor mutation status “ in the subgroup analysis, I² decreased to 38.7%, suggesting that this factor might be the source of heterogeneity. The heterogeneity test of AEs showed that χ²=11.79, df = 12, P = 0.4626. After stratification by patient group, dose, trial design, etc. in the subgroup analysis, the heterogeneity did not significantly improve. However, after stratification by “whether hematological toxicity occurred”, I² decreased to 36.2%, indicating that stratifying by the type of adverse events can reduce the heterogeneity of AEs results.

### Sensitivity analysis

Sensitivity analysis was performed for the important validity outcome measures (PFS and AEs) in the study. For PFS, only 1 Senaparib study was the least among the 18 literatures (13). After removing the mesh meta-analysis, SUCRA results showed: niraparib+bevacizumab(81.4%)> olaparib+cediranib(68.2%)>niraparib(59.8%)>rucaparib(53.2%)>fuzuloparib(46.7%)>olaparib (42.3%) > chemotherapy(35.6%) > placebo(8.72%). The results were consistent with the ranking order before exclusion, which proved that the results of mesh meta-analysis were reliable. For AEs, the results after deleting the same literature were consistent with the ranking order before deleting. All the above results indicate that the results of this study are stable. For AEs, even if any studies are excluded, the stability and reliability of the results will not be affected. These findings indicate that the results of this study are stable.

### Publication bias analysis (funnel chart test)

We used the funnel plot to qualitatively assess possible publication bias in the results of this study and small sample studies, and determined whether publication bias existed by observing the symmetry of the funnel plot. In the funnel plot, dots of various colors represent different clinical studies, and the closer the dots are to the X-axis, the small-sample benefit is indicated. In this study, the dispersion distribution in the funnel plot was symmetrical, and no evidence of possible small sample studies or publication bias was found, indicating that there was no significant publication bias affecting the results of the meta-analysis. Funnel plots were observed for survival (PFS) and AEs summary, suggesting accept-able publication bias (**Figures 4C, D**).

## Discussion

This study analyzed data from 15 randomized controlled trials across 18 publications, involving 6,416 Ovarian cancer patients and evaluating 9 therapeutic regimens. The findings demonstrate that PARP inhibitors combination therapy with anti-angiogenic agents shows superior clinical outcomes in ovarian cancer compared to PARP inhibitor monotherapy. Among the evaluated protocols, the niraparib-Bevacizumab combination exhibited the most pronounced progression-free survival (PFS) benefits, followed by the olaparib-cediranib regimen. Notably, the incidence of grade ≥3 adverse events (AEs) showed no statistically significant difference between monotherapy and combination treatment groups (P≥0.05). For bevacizumab, its anti-angiogenic mechanism does not rely on specific tumor mutations. Even if there is progression after the first-line treatment, it can still exert an effect by blocking the supply of tumor blood vessels. Studies have found that it is an effective treatment option in both platinum-resistant and platinum-sensitive recurrent ovarian cancer. For instance, some trials have confirmed the survival benefits and safety of its cross-line application, and it has been recommended for inclusion, which provides a reasonable treatment option for patients with recurrence (25, 26).

Therapeutic outcomes analysis revealed that combination therapy with PARP inhibitors and anti-angiogenic agents demonstrated superior clinical efficacy over PARP inhibitor monotherapy in ovarian cancer patients. SUCRA ranking suggested potential superiority of senaparib and relative inferiority of niraparib monotherapy. The findings confirm that PARP inhibitor-based combination therapy with anti-angiogenic agents represents a viable therapeutic strategy for ovarian cancer patients, aligning with the conclusions reported by Luo et al. (22).Our study advances existing evidence through the inclusion of two novel PARP inhibitors (fuzuloparib and senaparib), thereby strengthening the robustness of these findings through expanded pharmacological diversity. Regarding overall survival (OS) analysis, the limited number of eligible studies precluded network meta-analysis, thus no comparative evaluation was conducted for OS outcomes. Because OS data is crucial in oncology decision-making, and the improvement in progression-free survival (PFS) does not always translate into an increase in OS, therefore specific recommendations need to be provided for each treatment situation. Notably, a separate meta-analysis by Chen et al. (11) identified the olaparib-bevacizumab combination as demonstrating superior progression-free survival (PFS) benefits in both overall and non-BRCA mutant populations, showing partial discrepancies with our findings. This divergence likely stems from variations in the included studies across different analyses. These findings highlight the necessity for expanded clinical trials evaluating combination therapies involving PARP inhibitors and anti-angiogenic agents in ovarian cancer management, particularly to address current evidence inconsistencies arising from differences in the specific studies included.

This study found that the incidence of grade ≥3 adverse events (AEs) was highest with olaparib +cediranib and niraparib+bevacizumab among combination therapies. For olaparib+cediranib, the AEs rate was approximately 29.28%, with non-hematological toxicities such as hypertension (29.66%), fatigue (16.73%), and diarrhea (14.07%) being more common. In contrast, niraparib+ bevacizumab exhibited a distinct AEs profile, primarily characterized by anemia (15.0%), thrombocytopenia(10.0%), and hypertension (21.0%). Among PARP inhibitor monotherapies, Senaparib had the highest incidence of grade ≥3 AEs (63.8%), followed by niraparib (59.8%), Rucaparib (53.2%), Fuzuloparib (46.7%),and olaparib (42.3%).Hematological toxicities, including thrombocytopenia, anemia, and neutropenia, were predominant in monotherapy, with mortality events being rare (<0.5%). The study revealed no statistically significant differences in the incidence of grade ≥3 AEs between PARP inhibitor monotherapy and combination therapies (P≥0.05), consistent with the findings of Xu et al. (27) and prior network meta-analyses on PARP inhibitor safety profiles (28).

Our network meta-analysis offers several distinct advantages: (a) Methodological rigor was ensured through comprehensive database searches, including gray literature, and dual-reviewer verification to minimize publication bias. (b) This study represents the first network meta-analysis to evaluate the efficacy and safety of various PARP inhibitors, both as monotherapies and in combination with anti-angiogenic agents, across different stages of ovarian cancer treatment. By employing ranking probabilities, we were able to identify subtle differences between treatment groups. (c) Compared to previously published meta-analyses, our study encompasses five PARP inhibitors and two combination regimens, incorporating nearly all available clinical trial data. This extensive coverage of treatment options provides a more comprehensive analysis, significantly contributing to the standardization and optimization of ovarian cancer treatment protocols.

Meanwhile, compared with other studies, our research also has some common limitations: (a) In our study, the number of literatures was relatively insufficient, and ultimately only 18 literatures were included. (b) Although we conducted subgroup analyses, the information obtained was limited. In addition, the patients’ own conditions and the specific dosages in the treatment measures were not discussed. (c) Compared with previous network meta-analyses, although our study included more eligible studies, it inevitably overlooked some details in the studies.

## Conclusion

Offer ovarian cancer patients viable treatment options, such as niraparib combined with bevacizumab may have a better therapeutic effect in improving the progression-free survival period. And senaparib as monotherapy for more significant progression-free survival benefits, while reminding of potentially higher adverse events (grade≥3) to aid personalized choices. This study also provides guidance for the stage-specific and subtype-specific treatment of ovarian cancer, and emphasizes the need to conduct more high-quality long-term randomized controlled trials and large-scale multicenter studies to strengthen the evidence-based basis for clinical practice. Additionally, regulatory approval, biomarker status, and real-world tolerability will also affect the choice of treatment plans.

## Data Availability

The original contributions presented in the study are included in the article/**Supplementary Material**. Further inquiries can be directed to the corresponding authors.
