# Supplementary material for: Efficacy and safety of PARP inhibitors monotherapy or combination therapy with anti-angiogenics in ovarian cancer: a network meta-analysis
Source: Front Oncol. 2026 Jan 26;15:1713163. doi: 10.3389/fonc.2025.1713163 (PMC12883406; doi:10.3389/fonc.2025.1713163)
Supplement: Supplementary file 1 [file Supplementaryfile1.docx]

**Table 1-1. Search strategy on Pubmed.**

| **Search** | **Search terms** |
| --- | --- |
| #1 | ((Ovarian cancer[MeSH Terms]) OR (Ovarian Neoplasms[MeSH Terms])) OR (Carcinoma, Ovarian Epithelial[MeSH Terms]) |
| #2 | ((((((((((((Ovarian Neoplasm[Title/Abstract]) OR (Ovary Neoplasms[Title/Abstract])) OR (vary Neoplasm[Title/Abstract])) OR (Ovary Cancer[Title/Abstract])) OR (vary Cancers[Title/Abstract])) OR (Cancer of Ovary[Title/Abstract])) OR (Ovarian Cancer[Title/Abstract])) OR (Ovarian Cancers[Title/Abstract])) OR (Epithelial Carcinoma, Ovarian[Title/Abstract])) OR (Ovarian Epithelial Carcinomas[Title/Abstract])) OR (Ovarian Epithelial Cancer[Title/Abstract])) OR (Ovarian Cancer, Epithelial[Title/Abstract])) OR (Epithelial Ovarian Cancer[Title/Abstract]) |
| #3 | #1 OR #2 |
| #4 | (((((((PARP inhibitors) OR (olaparib)) OR (niraparib)) OR (rucaparib)) OR (veliparib)) OR (pamiparib)) OR (talazoparib)) OR (fluzoparib) |
| #5 | ((((((((((((Antiangiogenic drugs) OR (bevacizumab)) OR (pazopanib)) OR (nintedanib)) OR (cediranib)) OR (sorafenib)) OR (sunitinib)) OR (anlotinib)) OR (lenvatinib)) OR (regorafenib)) OR (erlotinib)) OR (vandetanib)) OR (trebananib) |
| #6 | (#4) AND (#5) |
| #7 | (#3) AND (#4) |
| #8 | (#3) AND (#6) |
| #9 | (#7) OR (#8) |

**Table 1-2. Search strategy on Web of science.**

| **Number** | **Search terms** |
| --- | --- |
| #1 | (((((((((((((ALL=(Ovarian cancer)) OR ALL=(Ovarian Neoplasms))OR ALL=( Carcinoma, Ovarian Epithelial)) OR ALL=(Ovary Cancer)) OR ALL=(vary Cancers)) OR ALL=(Cancer of Ovary)) OR ALL=(Ovarian Cancer)) OR ALL=(Ovarian Cancers)) OR ALL=( Epithelial Carcinoma, Ovarian)) OR ALL=(Ovarian Epithelial Carcinomas)) OR ALL=(Ovarian Epithelial Cancer)) OR ALL=( Ovarian Cancer, Epithelial)) OR ALL=( Epithelial Ovarian Cancer)) OR ALL=( pithelial Ovarian Carcinomas) |
| #2 | (((((((( ALL=( PARP inhibitors)) OR ALL=( olaparib)) OR ALL=( niraparib))OR ALL=( rucaparib)) OR ALL=( veliparib) OR ALL=( pamiparib) OR ALL=( talazoparib) OR ALL=( fluzoparib) |
| #3 | (((((((((((ALL= (Antiangiogenic drugs) OR ALL= (bevacizumab)) OR ALL= (pazopanib)) OR ALL= (nintedanib)) OR ALL= (cediranib)) OR ALL= (sorafenib)) OR ALL= (sunitinib)) OR ALL= (anlotinib)) OR ALL= (lenvatinib)) OR ALL= (regorafenib)) OR ALL= (erlotinib)) OR ALL= (vandetanib)) OR ALL= (trebananib) |
| #4 | #2 AND #3 |
| #5 | #1 AND #2 |
| #6 | #1 AND #4 |
| #7 | #5 OR #6 |

**Table 1-3. Search strategy on Corcrane.**

| **Number** | **Search terms** |
| --- | --- |
| #1 | (Ovarian Neoplasms):ti,ab,kw OR (Carcinoma, Ovarian Epithelial):ti,ab,kw OR (Ovarian Cancer):ti,ab,kw OR (Ovary Cancer):ti,ab,kw OR (Cancer of the Ovary):ti,ab,kw |
| #2 | (Epithelial Carcinoma, Ovarian):ti,ab,kw OR (Ovarian Epithelial Carcinomas):ti,ab,kw OR (Ovarian Epithelial Cancer):ti,ab,kw OR (Epithelial Ovarian Cancer):ti,ab,kw OR (Ovarian Cancer, Epithelial):ti,ab,kw |
| #3 | #1 OR #2 |
| #4 | (PARP inhibitors):ti,ab,kw OR (olaparib):ti,ab,kw OR (niraparib):ti,ab,kw OR (rucaparib):ti,ab,kw OR (veliparib):ti,ab,kw |
| #5 | (pamiparib):ti,ab,kw OR (talazoparib):ti,ab,kw OR (fluzoparib):ti,ab,kw |
| #6 | #4 OR #5 |
| #7 | (Antiangiogenic drugs):ti,ab,kw OR (bevacizumab):ti,ab,kw OR (pazopanib):ti,ab,kw OR (nintedanib):ti,ab,kw OR (cediranib):ti,ab,kw |
| #8 | (sorafenib):ti,ab,kw OR (sunitinib):ti,ab,kw OR (anlotinib):ti,ab,kw OR (lenvatinib):ti,ab,kw OR (regorafenib):ti,ab,kw |
| #9 | (erlotinib):ti,ab,kw OR (vandetanib):ti,ab,kw OR (trebananib):ti,ab,kw |
| #10 | #7 OR #8 OR #9 |
| #11 | #6 AND #10 |
| #12 | #3 AND #6 |
| #13 | #3 AND #11 |
| #14 | #12 OR #13 |

**Table 1-4. Search strategy on Embase.**

**.**

| **Number** | **Search terms** |
| --- | --- |
| #1 | 'ovarian neoplasms '/exp OR ' ovarian cancer ' OR (('ovarian'/exp OR ovarian) OR ('cancer'/exp OR cancer)) OR 'ovarian carcinoma ':ab,ti OR 'unilateral ovarian neoplasms':ab,ti OR ' recur ovarian cancer':ab,ti OR ' epithelial ovarian carcinoma ':ab,ti OR 'ovarian tumor':ab,ti OR 'ovarian tumors':ab,ti OR 'cancer of ovarian':ab,ti OR 'ovarian carcinoma':ab,ti |
| #2 | 'PARP inhibitors':ab,ti OR 'olaparib':ab,ti OR 'niraparib':ab,ti OR 'rucaparib':ab,ti OR  'veliparib ':ab,ti OR ' pamiparib ':ab,ti OR ' talazoparib ':ab,ti OR ' fluzoparib ':ab,ti |
| #3 | ' antiangiogenic drugs ':ab,ti OR ' bevacizumab ':ab,ti OR  ' pazopanib ':ab,ti OR ' nintedanib ':ab,ti OR ' cediranib ':ab,ti OR ' sorafenib ':ab,ti OR ' sunitinib ':ab,ti OR ' anlotinib ':ab,ti OR ' lenvatinib ':ab,ti OR ' regorafenib ':ab,ti OR ' erlotinib ':ab,ti OR ' vandetanib ':ab,ti OR ' trebananib ':ab,ti |
| #4 | #2 AND #3 |
| #5 | #1 AND #2 |
| #6 | #1 AND #4 |
| #7 | #5 AND #6 |
